# Adolescents, Young Adults, and Adults Continue to Use E-Cigarette Devices and Flavors Two Years after FDA Discretionary Enforcement

**DOI:** 10.3390/ijerph19148747

**Published:** 2022-07-18

**Authors:** Devin M. McCauley, Shivani Mathur Gaiha, Lauren Kass Lempert, Bonnie Halpern-Felsher

**Affiliations:** 1 Reach Lab, Department of Pediatrics, Division of Adolescent Medicine, Stanford University, Palo Alto, CA 94304, USA; dmm16@stanford.edu (D.M.M.); gaiha@stanford.edu (S.M.G.); llempert@stanford.edu (L.K.L.); 2 Center for Tobacco Control Research and Education, University of California, San Francisco, CA 94143, USA

**Keywords:** e-cigarettes, e-cigarette devices, flavors, adolescents, young adults, adults, Food and Drug Administration, menthol

## Abstract

This study assesses the use of e-cigarette devices and flavors using a large, cross-sectional survey of adolescents, young adults, and adults (N = 6131; ages 13–40 years old; M_age_ = 21.9) conducted from November to December 2021, 22 months after the FDA announced its prioritized enforcement policy against some flavored pod/cartridge-based e-cigarettes. We analyzed the patterns of use by age group: adolescents and young adults (AYAs) under 21 (minimum age of e-cigarette sales), young adults (21–24 years old), and adults (25–40 years old). The participants reported using e-cigarettes ever (44.2% < 21; 67.1% 21–24; 58.0% > 24), in the past 30 days (29.8% < 21; 52.6% 21–24; 43.3% > 24), and in the past 7 days (24.5% < 21; 43.9% 21–24; 36.5% > 24). Disposables were the most used e-cigarette device type across age groups (39.1% < 21; 36.9% 21–24; 34.5% > 24). Fruit, sweet, mint, and menthol flavors were popular across age groups; however, chi-squared tests for trends in proportions revealed age-related trends in past 30-day flavor use by device type. Findings suggest current AYA e-cigarette use may be higher than recorded by the NYTS 2021. The FDA, states, and localities should adopt more comprehensive restrictions on flavored e-cigarette products in order to reduce adolescent and young adult e-cigarette use.

## 1. Introduction

Over the last decade, e-cigarettes have become increasingly popular among adolescents and young adults (AYAs) [1,2,3], a trend attributable to marketing campaigns targeting young people, the availability of attractive flavors, and AYAs’ misperceptions of the nicotine levels, addictiveness, and real and potential health consequences of e-cigarettes [4,5,6,7,8,9]. With the introduction of JUUL in 2015, adolescent use of pod/cartridge-based e-cigarettes, in particular, began to increase substantially [2,10].

In 2019, national surveys provided further evidence for adolescents’ preference for flavored pod/cartridge-based e-cigarette devices [1] as well as for fruit and mint flavors relative to menthol and tobacco [11]. Citing these data, in January 2020 the FDA first announced that it would prioritize enforcement against the following products on the market that did not have premarket authorization: (1) flavored pod/cartridge-based e-cigarettes (with the exception of menthol- and tobacco-flavored), (2) e-cigarette products without adequate measures to prevent use by minors, and (3) products targeting minors or whose marketing is likely to promote use by minors [12]. Notably exempt from the FDA’s 2020 enforcement policy were disposable e-cigarette devices, which are discarded when the e-liquid is depleted rather than re-filled [13], other e-cigarettes such as “tank” or “mod” devices [14], and tobacco- and menthol-flavored pod/cartridge-based e-cigarettes [12].

Data collected following the FDA’s 2020 prioritized enforcement announcement illustrate a number of notable trends, including an increase in AYAs’ use of newer disposable-type e-cigarette devices and menthol flavors, as well as continued use of fruit, candy, and mint-flavored products, even in targeted pod/cartridge-based e-cigarettes [15,16,17]. These findings suggest that AYAs are gravitating toward exempted e-cigarette devices and flavors, yet also continue to access flavored pod/cartridge-based e-cigarettes targeted by the FDA [15]. Such patterns of use emphasize the need for the continued monitoring of evolving trends in the use of e-cigarette devices and flavors.

Subsequent national surveillance data suggest an unexpectedly low prevalence of adolescent e-cigarette use, with the 2021 National Youth Tobacco Survey (NYTS) indicating that only 11.3% of high school students and 2.8% of middle school students have used an e-cigarette in the past 30 days [18], down from 27.5% and 10.5%, respectively, as reported by the NYTS 2019 [1,19]. However, methodological changes in NYTS 2021 data collection due to the COVID-19 pandemic limit the interpretation of trends in adolescent e-cigarette use based on year-to-year comparisons of NYTS data [18]. Data from Monitoring the Future 2021 provide evidence for a higher prevalence of adolescents’ past 30-day e-cigarette use relative to the NYTS 2021 (i.e., 19.6% of 12th graders, 13.1% of 10th graders, and 7.6% of 8th graders) yet also indicate a lower prevalence relative to the 2019 data [20].

We do not yet have national data assessing the overall and specific e-cigarette devices and flavors used after adolescents have returned to in-person school and since COVID-19 restrictions have relaxed, leaving it unclear whether these transitions back to more normal school and social patterns have been accompanied by an increase in e-cigarette use. Further, there is a scarcity of data collected from adolescents, young adults, and adults within the same sample, making it difficult to draw clear and reliable comparisons in the use of e-cigarette devices and flavor types among different age groups. In addition to offering a comparison group for younger e-cigarette users, including adult e-cigarette users in study samples provides a more comprehensive assessment of patterns of use following the FDA’s targeted enforcement policy. Advancing our understanding of the similarities and differences in adolescent, young adult, and adult patterns of e-cigarette use may offer novel guidance for local, state, and federal policies aimed at mitigating the specific tobacco-related health risks faced by different age groups.

The current study builds upon recent investigations of e-cigarette use with data collected in November and December of 2021, 22 months after the January 2020 FDA guidance on enforcement priorities and 1 month after the FDA authorized its first set of e-cigarette products through the Premarket Tobacco Product Application (PMTA) pathway [12,21]. Additionally, data for the current study were collected six months after the NYTS 2021 assessments and therefore may capture more recent trends in e-cigarette use following adolescents’ return to in-person school. This study also expands upon other research by including three age groups: AYAs below the legal age of tobacco sales (13–20 years old), young adults over the legal age of tobacco sales (21–24 years old), and adults (25–40 years old) in one sample. In doing so, this study is uniquely positioned to detect age-related trends in e-cigarette device and flavor use, which may in turn offer novel guidance for policy and prevention. In particular, this study addresses the following research questions: (1) What percentage of AYAs under 21, young adults, and adults have ever used disposable, pod/cartridge-based, and other e-cigarettes (e.g., mods and tanks) in the past 30 days and the past 7 days, and do these percentages vary by sex?; (2) Which e-cigarette device types are most frequently used and in what combinations by these three age groups, and how do the use patterns differ across age groups?; (3) What flavor types are most used among AYAs under 21, young adult, and adult e-cigarette users?; (4) Are there age trends in past 30-day flavor use for disposable, pod/cartridge-based, and other e-cigarettes?; and (5) Which disposable and pod/cartridge-based e-cigarette brands are most popular among AYAs under 21, young adults, and adults?

## 2. Method

### 2.1. Procedure

We conducted a national, cross-sectional, anonymous online survey from 17 November to 15 December 2021, using convenience sampling. All survey participants were recruited by Qualtrics through online research panels. All panelists electing to participate provided assent/consent online before beginning the survey. We intentionally sampled participants aged 13 to 17 years, 18 to 20 years, and 21 to 40 years, representing adolescents, young adults below the legal age of tobacco sales, and young adults and adults, respectively. Qualtrics also balanced the sampling of sex and race/ethnicity to correspond to the latest US Census data. The final sample included 6131 participants. For more details on the data collection procedures, see [22]. This study was approved by the Institutional Review Board at Stanford University.

Participants self-reported sociodemographic information in the form of age, sex, race/ethnicity, and financial comfort. The final sample included 6131 participants, of whom *n* = 3633 (59.7%) were 13–20 years old (AYAs under the age of 21, the federal minimum legal age of sales for any nicotine or tobacco product in the United States), *n* = 1041 (17.0%) were 21–24 years old, and *n* = 1427 (23.3%) were 25–40 years old; *n* = 3454 (56.3%) were female. The majority of our sample was White, non-Hispanic (51.6%), followed by Hispanic or Latino (18.6%), African American or Black, non-Hispanic (13.7%), Asian, Native Hawaiian or Pacific Islander (5.2%), and other race or multiple races selected (4.1%). Some participants (6.8%) preferred not to disclose their race and ethnicity. Participants reported their financial comfort as ‘does not meet basic expenses’ (5.1%), ‘just meet expenses with nothing left over’ (18.2%), ‘meet needs with a little left over (27.0%), and ‘live comfortably’ (42.1%), with some participants indicating ‘prefer not to say’ (7.6%). See [22] for more sample information.

### 2.2. Measures

All study measures and items were pilot tested with assistance from 42 AYAs and adults.

#### 2.2.1. E-Cigarette Use: Ever Use, Past 30-Day Use, Past 7-Day Use, and Age at First Use

Participants were shown images of disposable, pod/cartridge-based, and other e-cigarette devices along with a description of each product. They were then asked, “Have you ever used any of these products in your entire life? (please select Yes OR No)” in relation to the following products: (a) Disposable nicotine vapes such as Puffbar or FOGG, even 1 or 2 puffs; (b) Pod-based nicotine vapes such as JUUL or Phix, even 1 or 2 puffs; and (c) Any other nicotine vape-like mods, even 1 or 2 puffs. Response options were not mutually exclusive. Participants who answered “yes” to having ever used products (a), (b), or (c) above were categorized as a nicotine e-cigarette ever user.

For the products where the respondents answered “yes”, the respondents were then asked to select a number from 0 to 30, “During the last 30 days, on about how many days did you use each of the following products? (Put zero if you did not use the product in the last 30 days).” Participants who selected any option other than zero for any products (a), (b), or (c) above were categorized as a past 30-day e-cigarette user.

For the e-cigarette device types where the respondents indicated past 30-day use, we then asked them to select a number from 0 to 7, “During the last 7 days, on about how many days did you use each of the following products? (Put a 0 if you did not use the product in the last 7 days)”. Participants who selected any option other than zero for products (a), (b), or (c) above were categorized as a past 7-day e-cigarette user.

#### 2.2.2. Most Used E-Cigarette Device Type

Participants indicated their most used e-cigarette device type by responding to the question, “Which best describes the type of vape you use/d most? (select one)” with 5 response categories: (1) Disposable vape-like Puff Bar; (2) Pod-based vape-like JUUL; (3) Other vape-like mod; (4) Non-nicotine vape; (5) Other (please describe); and (6) Don’t know.

#### 2.2.3. Flavor Types Used in E-Cigarettes: Ever Use, Past 30-Day Use, and Most Used in Past 30 Days

Ever-users of e-cigarettes (disposable, pod/cartridge-based, other) responded to the question “Which of these flavors or smells were in any [(disposable; pod-based; other) vape] that you used? (select all that apply)” by selecting from 19 answer choices presented in random order: (1) Flowers (e.g., rose, lavender, geranium, jasmine, hibiscus); (2) Essential oils (e.g., frankincense, tea tree); (3) Other plant extract (e.g., gingko, sandalwood); (4) Spice (e.g., clove, cinnamon, nutmeg); (5) Coffee or any related flavor (e.g., espresso, latte, cappuccino); (6) Tea; (7) Mint; (8) Menthol; (9) Ice; (10) Wintergreen; (11) Fruit (e.g., mango, lychee, cherry, blueberry, strawberry, watermelon, coconut); (12) Sweets or dessert flavors (e.g., crème or crème brûlée, caramel, vanilla, chocolate, ice cream, mud pie); (13) Alcohol (e.g., wine, bourbon, rum, brandy, tequila, whiskey, beer, mai-tai, daiquiri); (14) Candy; (15) Other beverage (e.g., Coca-Cola, etc.); (16) Unflavored; (17) Tobacco-flavored; (18) Don’t know/Don’t remember; and (19) Other, please specify (text-entry option). Flavor use was collapsed into types. Any use of flavor types (7), (8), (9), or (10) was categorized as “Mint/menthol flavor type,” and any use of flavor types (12) and (14) were categorized as “Sweet/dessert/candy flavor type”.

Next, ever-users of each e-cigarette device type (disposable, pod/cartridge-based, other) responded to the question, “In the past 30 days, which of these flavors or smells were in any [(disposable; pod-based; other) vape] that you used? (select all that apply)”. Finally, ever-users of e-cigarettes (disposable, pod/cartridge-based, other) responded to the question “In the PAST 30 DAYS, which flavor did you use the MOST in any [(disposable; pod-based; other) vape] (select one)”. Answer choices and flavor type collapsing were identical to those described above.

#### 2.2.4. E-Cigarette Brands

Past 30-day users of disposable e-cigarettes responded to the question “Which of these disposable vapes have you ever used? (select all that apply)” in relation to the following brands: (1) Puffbar; (2) PuffPlus; (3) VGOD Stig; (4) Posh; (5) Mojo; (6) Fogg; (7) Unicorn; (8) Halo; (9) Mule; (10) Dinner Lady Vape Pen Max; (11) Cali Bar; (12) Hyppe Bar; (13) SWFT; (14) DJI; (15) Flum; and (16) Other, please specify. Past 30-day users of pod/cartridge-based e-cigarettes responded to the question “Which of these pod-based vapes have you ever used? (select all that apply)” in relation to the following brands: (1) JUUL; (2) Phix; (3) Smok; (4) Logic; (5) Suorin; (6) Stizzy; (7) Blue; (8) VUSE; (9) NJoy; (10) Aspire; (11) Ijoy; (12) Xpod; (13) Apollo Brez; (14) VOOPOO; (15) Uwell; (16) Geek Vape; (17) Vaporesso; (18) Lost Vape; (19) Inokin; (20) Hyde; and (21) Other, please specify. Response options for both questions were: (1) Never used this brand; (2) Used, but NOT in the past 30 days; and (3) Used in the past 30 days.

### 2.3. Data Analysis

Frequencies and percentages of e-cigarette device and flavor use were calculated for the three age groups: AYAs under 21 (13–20 years), young adults (21–24 years), and adults (25–40 years), as well as for those under age 18 (i.e., 13–18). To assess e-cigarette use, we described frequencies and percentages of ever, past 30-day, and past 7-day use of any e-cigarette device, as well as for specific e-cigarette devices (disposable, pod/cartridge-based, and other e-cigarettes), overall, by age group, and between males and females. We also calculated the average number of days that past 30-day users reported using e-cigarette devices as well as the proportion of participants who used them on at least 20 of 30 days in the past 30 days and those who used them daily (defined as used 30 out of 30 days). Next, for device types and flavors used, we tabulated frequencies and percentages related to the most used e-cigarette devices and flavors among e-cigarette ever users, flavors used in the past 30 days, and most used flavor among past 30-day users across all device types. Percentages were calculated out of the total available responses. Data were missing in less than 4% of cases for the primary study variables. Responses to disposable and pod/cartridge-based e-cigarette brand types contained between 4 and 7% missing data, with these data presented in the Supplementary Materials.

To address the research questions related to age differences in e-cigarette devices and flavors, two-tailed chi-square tests for trends in proportions were then applied to detect statistically significant increases or decreases in proportions across the three age groups (AYAs under 21, young adults, and adults). Results were considered statistically significant at *p* < 0.05. SPSS v28 and R Studio v.2021.09.2 were used for all data preparation and statistical tests.

## 3. Results

### 3.1. E-Cigarette Use: Ever, Past 30-Day, and Past 7-Day Use by Age and Device Type

Table 1 provides information on e-cigarette use by device type and age category. Out of all the study participants, 51.3% reported having ever used any e-cigarette device, and 37.1%, 38.7%, and 31.0% reported having ever used disposable, pod/cartridge-based, and other e-cigarettes, respectively. An examination of the e-cigarette devices used by each age group revealed that among all 13–20-year-olds in the sample, disposable e-cigarettes had the highest use (33.8%), 21.7% reported using disposable devices in the past 30 days, and 17.0% reported using disposable devices in the past 7 days. Among the participants 13–17 years old in our sample, 32.9% had ever used any e-cigarettes, 25.1% had ever used disposables, 24.4% had ever used pod/cartridge-based devices, and 17.0% had ever used other e-cigarettes. The percentage of participants reporting past 30-day use among 13–17-year-olds was 20.6% for any e-cigarette, 15.6% for disposables, 12.6% for pod/cartridge, and 8.2% for other e-cigarettes.

Among all 21–24-year-olds in our sample, pod/cartridge-based e-cigarettes had the highest estimates of ever use (51.2%), 35.1% reported using pod/cartridge devices in the past 30 days, and 26.7% reporting using pod/cartridge-based devices in the past 7 days. Likewise for 25–40-year-olds, pod/cartridge-based e-cigarettes had the highest estimates of ever use (43.6%), 29.7% reported using pod/cartridge devices in the past 30 days, and 23.8% reported using pod/cartridge devices in the past 7 days. Chi-squared tests for trends in proportions indicated that the proportion of participants reporting ever, past 30-day, and past 7-day use of each e-cigarette device type significantly increased across age groups (*p*’s < 0.001).

Among the 13–20-year-old participants who used an e-cigarette in the past 30-days, 25.3% of disposable users used them on ≥20/30-days in the past 30 days, 21.8% of pod/cartridge-based users used them on ≥20/30-days in the past 30 days, 15.2% of other e-cigarette users used them on ≥20/30-days in the past 30 days, and 29.2% of any e-cigarette users used them on >20/30-days in the past 30 days. Among the 21–24-year-olds, the corresponding percentages having used an e-cigarette on 20 of the past 30 days were lower and were as follows: 19.0% (disposable), 12.5% (pod/cartridge-based), 12.0% (other e-cigarette), and 20.1% (any e-cigarette device); for the 25–40-year-olds, the numbers were 10.3% (disposable) 11.8% (pod/cartridge-based), 11.2% (other e-cigarette), and 16.2% (any e-cigarette device). Chi-squared tests for trends in proportions revealed that the proportion of past 30-day users reporting use on ≥20/30 days significantly declined as the age group increased for all e-cigarette devices except other e-cigarettes (*p*’s < 0.001).

The percentages of 13–20-year-old past 30-day users in our sample who qualified as daily users (i.e., indicated using a device on 30 out of the past 30 days) were 18.8% (disposable), 16.5% (pod/cartridge-based), 10.8% (other e-cigarette), and 21.8% (any e-cigarette device). The percentages of daily users among 21–24-year-olds were 14.4% (disposable), 10.0% (pod/cartridge-based), 7.2% (other e-cigarette), and 14.7% (any e-cigarette device); for 25–40-year-olds the percentages were 6.8% (disposable), 7.9% (pod/cartridge-based), 9.3% (other e-cigarette), and 12.0%, (any e-cigarette device). The proportion of past 30-day users reporting use on 30/30 days declined as the age group increased for all e-cigarette devices except other e-cigarettes (*p*’s < 0.001).

Among 13–20-year-old past 30-day users of each device, the average number of days used in the past 30 for disposable was M = 10.7, SD = 11.0, for pod/cartridge was M = 9.9, SD = 10.5, and for other e-cigarettes was M = 8.4, SD = 9.2. For 21–24-year-old past 30-day users of each device, the averages for the number of days used in the past 30 were as follows: disposable (M = 9.4, SD = 9.9), pod/cartridge (M = 7.8, SD = 8.7), and other e-cigarettes (M = 7.4, SD = 8.1). For 25–40-year-old past 30-day users of each device, the averages for the number of days used in the past 30 for each device were: other e-cigarettes (M = 7.1, SD = 8.5), disposable (M = 6.4, SD = 8.0), and pod/cartridge-based e-cigarettes (M = 6.8, SD = 8.3). Ever, past 30-day, and past 7-day e-cigarette use by sex is presented in Supplementary Table S4. Among the 13–20-year-old participants, a higher percentage of females relative to males reported the use of any e-cigarette device ever (*p* < 0.001), in the past 30 days (*p* = 0.002), and in the past 7 days (*p* = 0.0155). However, among 21–24-year-olds, a higher percentage of males relative to females reported the use of any e-cigarette device in the past 30 days (*p* = 0.004). Among the 25–40-year-old participants, a higher percentage of males relative to females reported the use of any e-cigarette device ever (*p* = 0.03), in the past 30 days (*p* < 0.001), and in the past 7 days (*p* < 0.001). See Supplementary Table S4 for additional results.

### 3.2. E-Cigarette Device Types across Age Groups

Table 2 depicts the e-cigarette device types that ever e-cigarette users reported as their most used devices ever. Disposable e-cigarettes were the most used device type among 13–20-year-olds (39.1%), 21–24-year-olds (36.9%), and 25–40-year-olds (34.5%). Chi-squared tests for trends in proportions revealed that the proportion of participants reporting disposable e-cigarettes as their most used device type declined across age categories ($\chi^{2}$ = 4.88, *p* = 0.027) as did the proportion of users selecting “don’t know” ($\chi^{2}$ = 7.17, *p* = 0.007). Conversely, the proportion of participants reporting “other vapes” as their most used device type increased across age categories ($\chi^{2}$ = 7.71, *p* = 0.005). The proportion of ever e-cigarette users selecting pod/cartridge-based e-cigarettes or non-nicotine e-cigarette devices as their most used device type did not demonstrate significant trends across age categories.

Table 3 depicts the device-type combinations used amongst past 30-day users of e-cigarettes. For 13-to-20-year-old past 30-day users of e-cigarettes, disposables only was the most common response (26.1%), followed by all three device types (i.e., disposables, pod/cartridge-based, and other; 21.5%). For 21–24-year-olds, the most common response was all three device types (25.1%), followed by disposables only (17.0%). For 25–40-year-olds, the most common response was all three device types (25.9%), followed by pod/cartridge-based only (17.5%).

Chi-squared tests for trends in proportions revealed that the proportion of participants reporting only disposable e-cigarette use in the past 30 days significantly decreased across age categories ($\chi^{2}$ = 54.56, *p* < 0.001). The proportion of participants reporting only other e-cigarette use ($\chi^{2}$ = 12.64, *p* < 0.001), pod/cartridge-based and other e-cigarettes only ($\chi^{2}$ = 10.187, *p* = 0.001), and the use of all three devices ($\chi^{2}$ = 4.66, *p* = 0.031) significantly increased across age categories.

### 3.3. E-cigarette Flavor Types

The examination of the flavors used across e-cigarette devices revealed that fruit was the most commonly reported flavor type ever used among 13–20-year-olds and 21–24-year-olds (59.0% < 21; 57.8% 21–24), ever used in the past 30 days (50.4% < 21; 50.9% 21–24), and the most used in the past 30 days (39.9% < 21; 38.8% 21–24). Sweet/dessert/candy was the most commonly reported flavor type among 25–40-year-olds ever (50.9%). Mint/menthol was the most commonly reported flavor type among 25–40-year-olds in the past 30 days (48.4%) and the most used in past 30 days (32.7%). Mint/menthol was the second most commonly reported flavor type among 13–20-year-olds for ever use (55.0%) and 13–20-year-olds and 21–24-year-olds for past 30-day use (44.8% < 21; 47.4% 21–24) and the most used in the past 30 days (29.6% < 21; 30.2% 21–24). Assessing mint and menthol separately indicated the following estimates of use by age groups: ever use of mint: 32.2% < 21; 24.0% 21–24; 21.5% > 24; ever use of menthol: 29.1% < 21; 25.7% 21–24; 26.4% > 24; past 30-day use of mint: 21.1% < 21; 17.8% 21–24; 18.9% > 24; and past 30-day use of menthol: 21.4% < 21; 20.7% 21–24; 24.6% > 24. Additional information about the flavors used across e-cigarette devices are available in Supplementary Table S1.

Table 4 depicts past 30-day use of flavor types by device type and age category among past 30-day users of each device type. For disposable e-cigarettes, fruit was the most commonly reported flavor type among 13–20-year-olds (48.5%) and 21–24-year-olds (47.0%) and mint/menthol was the most commonly reported flavor type among 25–40-year-olds (40.5%). Mint/menthol flavors were the second most commonly reported flavor type by 13–20-year-olds (35.5%), whereas sweet/dessert/candy flavors were the second most commonly reported flavor type by 21–24-year-olds (38.9%) and 25–40-year-olds (35.6%).

For disposable e-cigarettes, chi-squared tests for trends in proportions revealed that the proportion of past 30-day fruit flavor use ($\chi^{2}$ = 23.62, *p* < 0.001) and “don’t know” responses ($\chi^{2}$ = 5.75, *p* = 0.016) significantly decreased across age categories. The proportion of past 30-day use of flower flavor types ($\chi^{2}$ = 13.21, *p* < 0.001), alcohol flavor types ($\chi^{2}$ = 5.21, *p* = 0.022), unflavored ($\chi^{2}$ = 8.37, *p* = 0.004), tobacco ($\chi^{2}$ = 19.49, *p* = < 0.001), coffee ($\chi^{2}$ = 4.34, *p* = 0.037), and tea flavor types ($\chi^{2}$ = 16.99, *p* < 0.001) significantly increased across age categories. All other disposable e-cigarette flavor types did not demonstrate significant trends in proportions across age categories.

For pod/cartridge-based e-cigarettes, mint/menthol was the most commonly reported flavor type across age groups (40.6% < 21; 36.8% 21–24; 33.6% > 24). Fruit flavors were the second most commonly reported flavor type by 13–20-year-olds (30.0%) and 21–24-year-olds (34.3%) and sweet/dessert/candy flavors were the second most commonly reported flavor type by 25–40-year-olds (32.1%). Chi-squared tests for trends in proportions revealed that the proportion of past 30-day use of mint/menthol flavor types ($\chi^{2}$ = 5.36, *p* = 0.021), menthol flavor types ($\chi^{2}$ = 4.50, *p* = 0.034), and “don’t know” responses ($\chi^{2}$ = 4.46, *p* = 0.035) for the use of flavors in pod/cartridge-based e-cigarettes significantly declined across age categories. The proportion of past 30-day use of essential oil flavor types ($\chi^{2}$ = 12.94, *p* < 0.001), alcohol flavor types ($\chi^{2}$ = 4.12, *p* = 0.042), other beverage flavor types ($\chi^{2}$ = 8.08, *p* = 0.004), unflavored ($\chi^{2}$ = 5.92, *p* = 0.015), and tobacco ($\chi^{2}$ = 17.12, *p* < 0.001) increased across age categories. All other pod/cartridge-based e-cigarette flavor types did not demonstrate significant trends in proportions across age categories.

For other e-cigarettes, sweet/dessert/candy was the most commonly reported flavor type by 13–20-year-olds (32.7%) and 21–24-year-olds (36.6%) and mint/menthol was the most commonly reported flavor type by 25–40-year-olds (39.6%). Chi-squared tests for trends in proportions revealed that the proportion of past 30-day use of mint/menthol ($\chi^{2}$ = 9.65, *p* = 0.002), menthol ($\chi^{2}$ = 15.09, *p* < 0.001), alcohol ($\chi^{2}$ = 3.92, *p* = 0.048), tea ($\chi^{2}$ = 6.29, *p* = 0.012), and tobacco ($\chi^{2}$ = 11.24, *p* = < 0.001) flavor types significantly increased across age categories. All remaining e-cigarette flavor types did not demonstrate significant trends in proportions across age category.

### 3.4. E-Cigarette Brands

Among past 30-day disposable e-cigarette users, Puffbar (26.3% < 21; 32.1% 21–24, 37.0% > 24), PuffPlus (21.2% < 21; 28.7% 21–24, 30.0% > 24), and Hyppe Bar (14.2% < 21; 21.2% 21–24; 21.0% > 24) were the most commonly used disposable e-cigarette brands in the past 30 days. Among past 30-day pod/cartridge-based e-cigarette users, JUUL (26.3% < 21, 34.3% 21–24, 40.7% > 24), VUSE (28.0% < 21, 28.2% 21–24, 26.3% > 24), and Hyde (26.5% < 21, 19.1% 21–24, 22.8% > 24) were the most commonly used pod/cartridge-based e-cigarette brands used in the past 30 days. Chi-squared tests for trends in proportions indicated that the proportion of past 30-day users who had used each brand in the past 30 days significantly increased across age categories for all disposable e-cigarette brands and all pod/cartridge-based e-cigarette brands, except for Smok (*p* = 0.100), VUSE (*p* = 0.585), and Hyde (*p* = 0.118). More information on past 30-day use of disposable and pod/cartridge-based e-cigarette brands is available in Supplementary Tables S2 and S3.

## 4. Discussion

This cross-sectional study assesses patterns of e-cigarette device and flavor use almost two years after the FDA announced its prioritized enforcement against some flavored pod/cartridge-based e-cigarettes without premarket authorization in January 2020 [12], and since PMTA submissions by e-cigarette companies and authorizations have commenced [21]. Data were drawn from participants aged 13 to 40 years old, thus facilitating comparisons in e-cigarette use across distinct age groups and providing novel insights into age-related trends in the use of devices and flavors following changes in federal prioritization.

Our findings indicate a high proportion of people across all age groups reporting e-cigarette use and support prior research documenting the increased popularity of disposable devices, particularly among AYAs under 21 [13,15,16,17]. Notably, estimates of ever and past 30-day e-cigarette use among AYAs under 21 were higher in our study than the estimates reported among middle and high school students in the 2020 and 2021 National Youth Tobacco Surveys [18,19]. Specifically, in our study, we found that 44.2% of those aged 13–20 had ever used an e-cigarette and nearly 30% had used it in the past 30 days. When restricting the age range to 13–17-year-olds, we still found that 32.9% had ever used an e-cigarette, and 20.6% had used it at least once in the past 30 days. It should be noted that data for the NYTS were collected from January to March 2021, representing a time when many students were attending school remotely from home due to COVID-19 restrictions, which may have contributed to reduced access to and use of e-cigarettes [23]. Data from the current study were collected in November and December 2021 when many COVID-19 restrictions had been lifted and students returned to in-person learning with friends and classmates, which are also common sources for acquiring e-cigarettes [8,24]. Such factors may account for the large differences observed between the data reported here and those reported by the NYTS 2021 and emphasize the need for the continued monitoring of AYA e-cigarette use as federal policy and factors related to the COVID-19 pandemic continue to shape the access and availability of e-cigarette devices and flavors.

In addition to finding high estimates of ever and past 30-day e-cigarette use in our sample, our data indicate that a high proportion of past 30-day users across age groups was using them on 20 days or more out of the past 30. For example, among past 30-day disposable e-cigarette users, approximately one in four 13–20-year-olds, one in five 21–24-year-olds, and one in ten 25–40-year-olds reported using their disposable devices on at least 20 of the previous 30 days. These data make it clear that many recent e-cigarette users are engaging in frequent or even daily use, increasing their risk for nicotine addiction [25,26]. Furthermore, among all past 30-day users, AYAs under 21 were more likely to use disposable and pod/cartridge-based e-cigarettes frequently (i.e., 20 out of 30 days) or daily (i.e., 30 out of 30 days) compared to their older counterparts, highlighting young users as being particularly vulnerable to nicotine dependence and addiction [26]. These patterns also dispel the notions that young users are primarily experimenting with occasional e-cigarette use and support further policy action to prevent frequent use, which increases the risk of nicotine addiction.

We identified several additional age-related trends in participants’ use of e-cigarette device types. Notably, the proportion of e-cigarette users reporting disposables as (a) their *most* used device and (b) the *only* device they used in the past 30 days was highest among AYAs under 21 and declined in the older age categories. Conversely, the opposite trend emerged for other e-cigarettes (e.g., mods), which were more commonly used by adults relative to younger users, despite being less popular than disposable and pod/cartridge-based e-cigarettes overall. A higher proportion of adults reported other e-cigarettes as the first device they used relative to adolescents and young adults. These age trends may be explained by the timing at which the different e-cigarette devices entered the market. For example, disposable e-cigarette devices such as Puff Bar are among the most recent to be manufactured and sold [15,16], becoming widely available at a time when many of the youngest adolescents in the current study may have started using e-cigarettes. The popularity of disposables among AYAs under 21 is likely also because after the FDA adopted its 2020 enforcement policy, cheap disposables were available in kid-friendly flavors, whereas pod/cartridge-based e-cigarettes were not, making these disposable devices a popular alternative for young users [13]. On the other hand, JUUL (i.e., the most popular pod/cartridge-based e-cigarette) entered the market in 2015 and accounted for stark increases in e-cigarette use among high school students through 2019 [14,27], a period when many 21–24-year-olds in the current study would have been in high school. Other e-cigarettes, such as “mods” and “tanks,” entered the market as part of an even earlier generation of devices, which likely predated 13–20-year-olds’ e-cigarette initiations [14]. Furthermore, these particular devices are known for their larger sizes, which limit concealability [14], a salient factor for younger users striving to avoid detection by adults [28,29].

Despite the recent popularity of disposable e-cigarette devices, it should be noted that in our sample, over 40% of past 30-day users in each age group reported using disposable e-cigarettes in combination with other device types (e.g., pod/cartridge and other) and over 20% reported that they had used disposables, pod/cartridge-based, and other e-cigarettes within the past 30 days. These findings highlight that for many e-cigarette users, including AYAs under 21, the rise in popularity of disposable devices does not translate to the exclusive use of these devices. Such data suggest the need to regulate all e-cigarette devices rather than one device type or one brand at a time.

Several age-related trends emerged when evaluating flavor use in e-cigarette devices. First, although flavor use was prevalent across age groups, a significantly lower proportion of e-cigarette users under the age of 21 reported using unflavored and tobacco-flavored disposable and pod/cartridge-based e-cigarette devices relative to their young adult and adult counterparts. This finding further supports the notion that youth, in particular, are drawn to flavors [6,30]. However, the FDA’s recent authorization of tobacco-flavored e-cigarettes including VUSE, Logic, and NJOY [31] is concerning as it remains possible that young users may misinterpret the FDA’s authorization to mean these products are safe or harmless and, as a result, initiate with authorized devices in the absence of flavored options. Also, many of these products are promoted together with their menthol-flavored counterparts, which have not yet been authorized but remain on the market.

Our results on the use of flavors in pod/cartridge-based e-cigarettes indicate that e-cigarette users continue to access and use popular pod/cartridge flavors targeted by the FDA, such as fruit and mint [12,32]. In addition, many pod/cartridge-based e-cigarette past 30-day users under 21 are now using menthol, a flavor exempted by the FDA’s targeted policy based on older reports of the low prevalence of use among adolescents [11,12]. In fact, in this study, a higher proportion of AYA under 21 who were past 30-day users of pod/cartridge-based e-cigarettes reported using menthol relative to their young adult and adult counterparts. Prior research indicates that menthol flavors provide a cooling sensation that reduces harshness and facilitates nicotine intake [4,33] and that young adults who initiate e-cigarette use with menthol flavors are at greater risk of more frequent use and nicotine dependence [34]. These factors highlight the rising popularity of menthol among AYAs and provide further evidence that the FDA should prohibit menthol as a flavor in e-cigarettes in addition to cigarettes and cigars.

Distinct trends emerged when evaluating flavors used in disposable e-cigarettes. In particular, AYAs’ past 30-day use of fruit flavors was particularly common in disposable e-cigarettes, which were exempt from the FDA’s enforcement priorities, relative to restricted pod/cartridge-based, which were subject to the FDA’s flavor policy. Furthermore, a higher proportion of disposable e-cigarette users under the age of 21 reported the use of fruit flavors relative to their adult counterparts. Collectively, these findings on flavor use may be the result of the FDA’s targeted restriction of flavored pod/cartridge devices, which left (a) disposable devices as an attractive source for fruit, candy, and mint flavors, and (b) menthol as an unrestricted yet attractive flavor option for pod/cartridge devices. Appealing flavors are one of the most salient factors in adolescent e-cigarette use [30,35]; therefore, policies that selectively restrict flavors in some e-cigarette devices may only precipitate a shift toward unrestricted alternatives rather than curtailing overall use.

## 5. Limitations and Future Directions

This study had a number of limitations that should be addressed in future research. First, data were drawn from a convenience sample, limiting generalizability to the entire population of adolescent, young adult, and adult e-cigarette users in the United States. However, our findings were drawn from a large sample size and the sex and race/ethnicity of study participants were balanced to reflect US Census data. It should also be noted that the results may not generalize to other countries, which are likely to have distinct attitudes, legal conditions, and policy developments related to e-cigarette use.

Additionally, this study was cross-sectional in design and therefore does not capture within-person changes in e-cigarette device and flavor use resulting from the FDA-targeted restrictions. Longitudinal studies would provide novel insights into intra-individual changes in e-cigarette use in response to federal policy and factors related to the COVID-19 pandemic. Furthermore, age categories in the current study reflect broad age ranges relevant to developmental phases and policy, and the use of devices and flavors is likely to vary within each group as well as by other important demographic factors such as race/ethnicity, sex, LGBTQ+ status, and geographic region. Future research may apply different methodologies to evaluate and detect more subtle age differences in e-cigarette use as well as moderation by key demographic factors (e.g., time-varying effect modeling) [36]. For example, our data indicate sex differences in estimates of ever and past 30-day e-cigarette use and that these sex differences vary by age group. Estimates of e-cigarette use were higher among female relative to male 13–20-year-old participants; however, this trend was reversed among the 25–40-year-old participants. Future research should further explore age-related sex differences in perceptions of risks and benefits, flavor preferences, addiction, points of access, and reasons for use. Progress in this domain may facilitate more tailored prevention strategies. Similarly, future research should more closely examine how patterns of e-cigarette use following the FDA’s 2020 prioritized enforcement may vary based on geographic region and in response to local attitudes and policies such as flavor bans [37].

Finally, disposable users across age groups reported that Puff Bar and Puff Plus, which claim to use synthetic rather than tobacco-based nicotine, were the most used disposable e-cigarette brand. Though synthetic nicotine is now regulated along with tobacco-based nicotine, consumers’ perceptions of synthetic nicotine, reasons for using synthetic nicotine, as well as potential health consequences and addictive properties are not yet fully understood and thus represent an avenue for future research.

## 6. Conclusions

The study findings indicate a high proportion of e-cigarette use among AYAs under 21, young adults, and adults, and suggest that current AYA e-cigarette use appears to be higher than recorded by the NYTS 2021. Notably, a high percentage of adolescents and young adults are using e-cigarettes despite the FDA’s 2020 prioritized enforcement against some e-cigarette devices and flavors previously observed as the most popular among young users, which suggests the ineffectiveness of this limited policy. Exempted flavored disposable e-cigarettes and menthol pod/cartridge e-cigarettes are particularly popular, suggesting that young users have turned to unrestricted products that allow them to continue accessing attractive flavors. Conversely, a higher percentage of adult users reported using unflavored and tobacco-flavored e-cigarette devices relative to users in younger age categories. These data along with other recent studies [15,17] strongly indicate that selective policy action is unlikely to effectively address the problem of adolescent e-cigarette use, particularly as tobacco companies continue to develop new products and marketing tactics to maintain profits and circumvent regulations [38,39]. Given the limited effectiveness of e-cigarettes as adult smoking-cessation devices [40,41] and the role of flavors in promoting adolescent e-cigarette use [6,30,34], the FDA should adopt more comprehensive policies that prohibit all flavors, including menthol, in all types of e-cigarette devices to help achieve its stated goal of stopping the public health crisis of adolescent e-cigarette use [12]. Furthermore, states and localities may consider enacting policies to limit or ban e-cigarette flavors, a strategy that has been associated with reductions in e-cigarette retail sales at the city level [37,42]. It should be noted, however, that local policies that restrict retail sales of flavored e-cigarettes may not prevent underage access through alternative sources, such as online purchases or social sources such as friends [43]. Consequently, the FDA, states, and localities should not only enforce manufacturers and retailers who sell products that violate existing federal, state, and local regulations, but also more comprehensively address adolescent e-cigarette use.

## Figures and Tables

**Table 1:** Ever, past 30-, and past 7-day e-cigarette use by age category and device type, no. (%).

		Age Category			*p*-Value
	Total (*N* = 6131)	13–20 (*N* = 3663)	21–24 (*N* = 1041)	25–40 (*N* = 1427)	
**Ever Use**					
Any E-cigarette	3137 (51.3)	1614 (44.2)	696 (67.1)	827 (58.0)	<0.001
Disposables	2257 (37.1)	1227 (33.8)	488 (47.5)	542 (38.4)	<0.001
Pod/cartridge-based	2345 (38.7)	1206 (32.2)	526 (51.2)	613 (43.6)	<0.001
Other E-cigarettes	1879 (31.0)	923 (25.5)	436 (42.5)	520 (37.1)	<0.001
**E-cigarette Use in the Past 30 Days**					
Any E-cigarette	2256 (36.9)	1092 (29.8)	546 (52.6)	618 (43.3)	<0.001
Disposables	1502 (24.7)	787 (21.7)	347 (33.8)	368 (26.0)	<0.001
Pod/cartridge-based	1419 (23.4)	641 (17.7)	361 (35.1)	417 (29.7)	<0.001
Other E-cigarettes	1122 (18.5)	474 (13.1)	292 (28.5)	356 (25.4)	<0.001
**E-cigarette Use on ≥20 Days in Past 30 Days among Past 30-Day Users**					
Any E-cigarette	529 (23.4)	319 (29.2)	110 (20.1)	100 (16.2)	<0.001
Disposables	303 (20.2)	199 (25.3)	66 (19.0)	38 (10.3)	<0.001
Pod/cartridge-based	234 (16.5)	140 (21.8)	45 (12.5)	49 (11.8)	<0.001
Other e-cigarettes	147 (13.1)	72 (15.2)	35 (12.0)	40 (11.2)	0.086
**Daily E-cigarette Use (i.e., 30 out of 30 days) among Past 30-Day Users**					
Any E-cigarette	392 (17.4)	238 (21.8)	80 (14.7)	74 (12.0)	<0.001
Disposables	223 (14.8)	148(18.8)	50 (14.4)	25 (6.8)	<0.001
Pod/cartridge-based	175 (12.3)	106 (16.5)	36 (10.0)	33 (7.9)	<0.001
Other e-cigarettes	105 (9.4)	51 (10.8)	21 (7.2)	33 (9.3)	0.404
**E-cigarette Use in the Past 7 Days**					
Any E-cigarette	1875 (30.6)	899 (24.5)	456 (43.9)	520 (36.5)	<0.001
Disposables	1186 (19.5)	617 (17.0)	281 (27.3)	288 (20.4)	<0.001
Pod/cartridge-based	1095 (18.1)	486 (13.4)	274 (26.7)	335 (23.8)	<0.001
Other e-cigarettes	847 (14.0)	350 (9.7)	225 (22.0)	272 (19.4)	<0.001

Note. Responses are not mutually exclusive: participants could report use for multiple e-cigarette devices and were counted for each device they used. *p*-values (<0.05) indicate statistically significant trends in proportions across three age categories. Percentages calculated out of all available responses. Missing data accounted for <2% of responses.

**Table 2:** Most used device type among ever users of e-cigarettes by age category, no. (%).

Device Type	Age Category				*p*-Value
	Total (*N* = 3069)	13–20 (*N* = 1580)	21–24 (*N* = 685)	25–40 (*N* = 804)	
Disposable	1147 (37.4)	**617 (39.1)**	**253 (36.9)**	**277 (34.5)**	0.027
Pod/cartridge-based	975 (31.8)	476 (30.1)	234 (34.2)	265 (33.0)	0.103
Other e-cigarettes	180 (5.9)	**76 (4.8)**	**43 (6.3)**	**61 (7.6)**	0.005
Non-nicotine	349 (11.4)	163 (10.3)	89 (13.0)	97 (12.1)	0.134
Other response	69 (2.2)	36 (2.3)	13 (1.9)	20 (2.5)	0.835
Don’t Know	349 (11.4)	**212 (13.4)**	**53 (7.7)**	**84 (10.4)**	0.007

Note. Bolded rows indicate statistically significant trends in proportions across age categories at *p* < 0.05. Percentages calculated out of all available responses. Missing data accounted for <3% of responses.

**Table 3:** Combinations of different e-cigarette devices used among past 30-day users, no. (%).

Device Type Combination	Age Category				*p*-Value
	Total (*N* = 2256)	13–20 (*N* = 1092)	21–24 (*N* = 546)	25–40 (*N* = 618)	
Disposable only	450 (19.9)	**285 (26.1)**	**93 (17.0)**	**72 (11.7)**	<0.001
Pod-based only	355 (15.7)	156 (14.3)	91 (16.7)	108 (17.5)	0.069
Other only	196 (8.7)	**76 (7.0)**	**45 (8.2)**	**75 (12.1)**	<0.001
Disposable & Pod-based only	329 (14.6)	177 (16.2)	70 (12.8)	82 (13.3)	0.068
Disposable & other only	191 (8.5)	90 (8.2)	47 (8.6)	54 (8.7)	0.712
Pod-based & other only	203 (9.0)	**73 (6.7)**	**63 (11.5)**	**67 (10.8)**	0.001
Disposable & Pod-based & Other	532 (23.6)	235 (21.5)	137 (25.1)	160 (25.9)	0.031

Note. Bolded rows indicate statistically significant trends in proportions across age categories at *p* < 0.05.

**Table 4:** Past 30-day flavor types used in different e-cigarette devices by age category, no. (%).

	Disposable (*N* = 1502)			Pod-Based (*N* = 1419)			Other (*N* = 1122)		
	13–20 (*N* = 787)	21–24 (*N* = 347)	25–40 (*N* = 368)	13–20 (*N* = 641)	21–24 (*N* = 361)	25–40 (*N* = 417)	13–20 (*N* = 474)	21–24 (*N* = 292)	25–40 (*N* = 356)
Sweet/dessert/candy ^a^	258 (32.8)	135 (38.9)	131 (35.6)	187 (29.2)	110 (30.5)	134 (32.1)	155 (32.7)	107 (36.6)	122 (34.3)
Sweets or dessert flavors	182 (23.1)	102 (29.4)	94 (25.5)	137 (21.4)	88 (24.4)	98 (23.5)	110 (23.2)	83 (28.4)	85 (23.9)
Candy	121 (15.4)	60 (17.3)	60 (16.3)	83 (12.9)	43 (11.9)	57 (13.7)	60 (12.7)	40 (13.7)	61 (17.1)
Fruit	**382 (48.5)**	**163 (47.0)**	**119 (32.3)**	192 (30.0)	124 (34.3)	124 (29.7)	152 (32.1)	91 (31.2)	98 (27.5)
Mint/menthol ^b^	279 (35.5)	121 (34.9)	149 (40.5)	**260 (40.6)**	**133 (36.8)**	**140 (33.6)**	**139 (29.3)**	**101 (34.6)**	**141 (39.6)**
Mint	137 (17.4)	46 (13.3)	52 (14.1)	98 (15.3)	48 (13.3)	51 (12.2)	46 (9.7)	30 (10.3)	40 (11.2)
Menthol	108 (13.7)	44 (12.7)	64 (17.4)	**143 (22.3)**	**64 (17.7)**	**72 (17.3)**	**43 (9.1)**	**35 (12.0)**	**65 (18.3)**
Wintergreen	69 (8.8)	39 (11.2)	44 (12.0)	48 (7.5)	45 (12.5)	39 (9.4)	48 (10.1)	38 (13.0)	39 (11.0)
Ice	104 (13.2)	46 (13.3)	46 (12.5)	65 (10.1)	34 (9.4)	32 (7.7)	47 (10.0)	35 (12.0)	34 (9.6)
Coffee	**84 (10.7)**	**49 (14.1)**	**54 (14.7)**	68 (10.6)	48 (13.3)	58 (13.9)	64 (13.5)	53 (18.2)	64 (18.0)
Flowers	**70 (8.9)**	**51 (14.7)**	**58 (15.8)**	69 (10.8)	49 (13.6)	59 (14.1)	56 (11.8)	40 (13.7)	35 (9.8)
Essential oils	65 (8.3)	39 (11.2)	34 (9.2)	**42 (6.6)**	**48 (13.3)**	**54 (12.9)**	39 (8.2)	35 (12.0)	28 (7.9)
Alcohol	**86 (10.9)**	**49 (14.1)**	**57 (15.5)**	**78 (12.2)**	**63 (17.5)**	**68 (16.3)**	**61 (12.9)**	**71 (24.3)**	**62 (17.4)**
Other Beverage	92 (11.7)	33 (9.5)	37 (10.1)	**33 (5.1)**	**36 (10.0)**	**40 (9.6)**	38 (8.0)	25 (8.6)	28 (7.9)
Unflavored	**39 (5.0)**	**29 (8.4)**	**34 (9.2)**	**39 (6.1)**	**35 (9.7)**	**42 (10.1)**	31 (6.5)	25 (8.6)	22 (6.2)
Spice	49 (6.2)	32 (9.2)	33 (9.0)	47 (7.3)	44 (12.2)	44 (10.6)	43 (9.1)	34 (11.6)	35 (9.8)
Other plant extract	50 (6.4)	34 (9.8)	30 (8.2)	56 (8.7)	41 (11.4)	38 (9.1)	47 (9.9)	37 (12.7)	38 (10.7)
Tea	**28 (3.6)**	**11 (3.2)**	**36 (9.8)**	36 (5.6)	24 (6.6)	28 (6.7)	**18 (3.8)**	**26 (8.9)**	**28 (7.9)**
Tobacco	**54 (6.9)**	**32 (9.2)**	**56 (16.1)**	**51 (8.0)**	**31 (8.6)**	**68 (16.3)**	**37 (7.8)**	**39 (13.4)**	**54 (15.2)**
Don’t know/remember	**64 (8.1)**	**26 (7.5)**	**15 (4.1)**	**70 (10.9)**	**30 (8.3)**	**30 (7.2)**	59 (12.4)	25 (8.6)	32 (9.0)
Other	20 (2.5)	14 (4.0)	12 (3.3)	24 (3.7)	18 (5.0)	17 (4.1)	26 (5.5)	8 (2.7)	11 (3.1)

Note. All flavor types reported (not mutually exclusive categories of use); ^a^ ‘sweet/dessert/candy’ flavor types show any use of sweet, dessert, or candy flavors; ^b^ ‘Mint/menthol’ flavor types show any use of mint, wintergreen, menthol, or ice flavors. Bolded rows within each device type indicate statistically significant trends in proportions of flavor use at *p* < 0.05.

## Data Availability

Study data are available upon request from the corresponding author.

## Supplementary Materials

The following supporting information can be downloaded at: https://www.mdpi.com/article/10.3390/ijerph19148747/s1, Table S1: E-cigarette flavor types used by age category (Ever, Past 30 Days, Most used in Past 30 Days), no. (%); Table S2: Past 30-day disposable e-cigarette brands used among past 30-day users of disposable e-cigarette devices, no. (%); Table S3: Past 30-day pod/cartridge-based e-cigarette brands used among past 30-day users of pod/cartridge-based e-cigarette devices, no. (%); Table S4: Ever, past 30-, and past 7-day e-cigarette use by gender, age category, and device type, no. (%).
