# Supplementary material for: Adolescents, Young Adults, and Adults Continue to Use E-Cigarette Devices and Flavors Two Years after FDA Discretionary Enforcement
Source: Int J Environ Res Public Health. 2022 Jul 18;19(14):8747. doi: 10.3390/ijerph19148747 (PMC9322506; doi:10.3390/ijerph19148747)
Supplement: Supplementary file 1 [file ijerph-19-08747-s001.zip › ijerph-1775403-supplementary.pdf]

Table S1. E-cigarette flavor types used by age category (Ever, Past 30 Days, Most used in Past 30 Days), no. (%).

	**Ever Use (N = 3137)**			**Past 30 Days (N = 2256)**			**Most used in the Past 30 Days (N = 2256)**		
	**13-20 (N = 1,614)**	**21-24 (N = 696)**	**25-40 (N = 827)**	**13-20 (N = 1,092)**	**21-24 (N = 546)**	**25-40 (N = 618)**	**13-20 (N = 1,092)**	**21-24 (N = 546)**	**25-40 (N = 618)**
Sweet/dessert/candy [a]	829 (51.4)	374 (53.7)	421 (50.9)	461 (42.2)	259 (47.4)	277 (44.8)	269 (24.6)	155 (28.4)	179 (29.0)
Sweets or dessert flavors	645 (40.0)	301 (43.2)	312 (37.7)	338 (31.0)	200 (36.6)	209 (33.8)	195 (17.9)	116 (21.2)	136 (22.0)
Candy	466 (28.9)	190 (27.3)	226 (27.3)	225 (20.6)	117 (21.4)	137 (22.2)	101 (9.2)	52 (9.5)	72 (11.7)
Fruit	**953 (59.0)**	**402 (57.8)**	365 (44.1)	**550 (50.4)**	**278 (50.9)**	250 (40.5)	**436 (39.9)**	**212 (38.8)**	200 (32.4)
Mint/menthol[b]	887 (55.0)	372 (53.4)	410 (49.6)	489 (44.8)	259 (47.4)	**299 (48.4)**	323 (29.6)	165 (30.2)	**202 (32.7)**
Mint	520 (32.2)	167 (24.0)	178 (21.5)	230 (21.1)	97 (17.8)	117 (18.9)	118 (10.8)	40 (7.3)	69 (11.2)
Menthol	469 (29.1)	179 (25.7)	218 (26.4)	234 (21.4)	113 (20.7)	152 (24.6)	154 (14.1)	66 (12.1)	103 (16.7)
Wintergreen	298 (18.5)	141 (20.3)	157 (19.0)	145 (13.3)	98 (17.9)	98 (15.9)	57 (5.2)	46 (8.4)	48 (7.8)
Ice	400 (24.8)	137 (19.7)	125 (15.1)	172 (15.8)	96 (17.6)	89 (14.4)	77 (7.1)	53 (9.7)	43 (7.0)
Coffee	273 (16.9)	166 (23.9)	189 (22.9)	171 (15.7)	116 (21.2)	137 (22.2)	99 (9.1)	63 (11.5)	85 (13.8)
Flowers	241 (14.9)	161 (23.1)	168 (20.3)	160 (14.7)	113 (20.7)	119 (19.3)	83 (7.6)	65 (11.9)	69 (11.2)
Essential oils	210 (13.0)	117 (16.8)	124 (15.0)	129 (11.8)	103 (18.9)	96 (15.5)	58 (5.3)	59 (10.8)	50 (8.1)
Alcohol	270 (16.7)	162 (23.3)	164 (19.8)	180 (16.5)	142 (26.0)	134 (21.7)	100 (9.2)	89 (16.3)	88 (14.2)
Other Beverage	249 (15.4)	132 (19.0)	127 (15.4)	140 (12.8)	77 (14.1)	90 (14.6)	65 (6.0)	40 (7.3)	46 (7.4)
Unflavored	175 (10.8)	100 (14.4)	109 (13.2)	95 (8.7)	76 (13.9)	80 (12.9)	32 (2.9)	26 (4.8)	35 (5.7)
Spice	199 (12.3)	101 (14.5)	126 (15.2)	121 (11.1)	83 (15.2)	90 (14.6)	62 (5.7)	44 (8.1)	53 (8.6)
Other plant extract	182 (11.3)	101 (14.5)	98 (11.9)	131 (12.0)	98 (17.9)	93 (15.0)	65 (6.0)	49 (9.0)	47 (7.6)
Tea	122 (7.6)	55 (7.9)	89 (10.8)	72 (6.6)	48 (8.8)	72 (11.7)	29 (2.7)	25 (4.6)	41 (6.6)
Tobacco	289 (17.9)	117 (16.8)	184 (22.2)	121 (11.1)	90 (16.5)	136 (22.0)	52 (4.8)	45 (8.2)	85 (13.8)
Don't know/remember	265 (16.4)	91 (13.1)	103 (12.5)	161 (14.7)	63 (11.5)	61 (9.9)	135 (12.4)	47 (8.6)	47 (7.6)
Other	66 (4.1)	32 (4.6)	26 (3.1)	63 (5.8)	36 (6.6)	34 (5.5)	11 (1.0)	7 (1.3)	5 (0.8)

Note. All flavor types reported (not mutually exclusive categories of use); a. 'sweet/dessert/candy' flavor types show any use of sweet, dessert, or candy flavors; b. 'Mint/menthol' flavor type shows any use of mint, wintergreen, menthol, or ice flavors. Bold indicates flavors used most often within each age category.

Table S2. Past 30-day disposable e-cigarette brands used among past 30-day users of disposable e-cigarette devices, no. (%).

	**Total Sample**	**Age Category**			***p*-Value**
		**13-20**	**21-24**	**25-40**	
**Disposable e-cigarette brand**					
Puffbar	437 (30.2)	201 (26.3)	107 (32.1)	129 (37.0)	<0.001
PuffPlus	357 (25.1)	158 (21.2)	95 (28.7)	104 (30.0)	<0.01
VGOD Stig	187 (13.0)	61 (8.1)	40 (12.1)	86 (24.7)	<0.001
Posh	238 (16.5)	90 (11.9)	63 (18.8)	85 (25.4)	<0.001
Mojo	196 (13.7)	70 (9.3)	48 (14.3)	78 (22.7)	<0.001
Fogg	231 (16.1)	90 (11.9)	62 (18.6)	79 (22.9)	<0.001
Unicorn	176 (12.3)	61 (8.1)	44 (13.2)	71 (20.6)	<0.001
Halo	199 (9.6)	75 (6.5)	50 (10.8)	74 (16.4)	<0.001
Myle	186 (13.0)	62 (8.2)	45 (13.4)	79 (22.8)	<0.001
Dinner Lady Vape Pen Max	182 (12.8)	64 (8.6)	45 (13.4)	73 (21.2)	<0.001
Cali Barr	229 (15.9)	86 (11.4)	60 (17.9)	83 (23.8)	<0.001
Hyppe Bar	251 (17.5)	107 (14.2)	71 (21.2)	73 (21.0)	<0.01
SWFT	190 (13.2)	73 (9.7)	50 (14.9)	67 (19.2)	<0.001
DJI	183 (12.8)	60 (8.0)	50 (15.0)	73 (21.0)	<0.001
Flum	218 (15.2)	76 (10.1)	64 (19.1)	78 (22.5)	<0.001

Note. Percentages calculated out of all available responses. Missing data accounted for between 4% and 5% of each response and were omitted. Brand responses are not mutually exclusive. P-values (<0.05) indicate statistically significant trends in proportions across three age categories.

Table S3. Past 30-day pod/cartridge-based e-cigarette brands used among past 30-day users of pod/cartridge-based e-cigarette devices, no. (%).

	Total Sample	Age Category			*p*-Value
		13-20	21-24	25-40	
**Pod/cartridge-based e-cigarette brand**					
JUUL	435 (32.6)	157 (26.3)	116 (34.3)	162 (40.7)	<0.001
Phix	179 (13.5)	56 (9.4)	50 (14.7)	73 (18.6)	<0.001
Smok	287 (21.7)	125 (20.9)	60 (18.0)	102 (25.9)	0.100
Logic	195 (14.7)	58 (9.8)	58 (17.1)	79 (20.2)	<0.001
Suorin	235 (16.9)	76 (12.8)	62 (18.1)	87 (22.8)	<0.001
Stiizy	206 (15.5)	67 (11.3)	49 (14.4)	90 (22.9)	<0.001
Blu	257 (19.3)	80 (13.4)	67 (19.8)	110 (27.6)	<0.001
VUSE	366 (27.5)	167 (28.0)	96 (28.2)	103 (26.3)	0.585
NJoy	240 (18.1)	80 (13.4)	59 (17.4)	101 (25.7)	<0.001
Aspire	192 (14.4)	59 (10.0)	51 (15.0)	82 (20.6)	<0.001
Ijoy	194 (14.6)	60 (10.1)	58 (17.0)	76 (19.2)	<0.001
Xpod	195 (14.6)	66 (11.0)	54 (15.8)	75 (18.8)	<0.001
Apollo Brez	173 (13.1)	55 (9.3)	46 (13.4)	72 (18.4)	<0.001
VOOPOO	201 (6.3)	63 (10.6)	62 (18.2)	76 (19.2)	<0.001
Uwell	200 (15.0)	66 (11.1)	53 (15.5)	81 (20.6)	<0.001
Geek Vape	175 (13.2)	59 (9.9)	50 (14.7)	66 (16.9)	<0.001
Vaporesso	227 (17.1)	68 (11.5)	65 (19.0)	94 (24.0)	<0.001
Lost Vape	189 (14.3)	58 (9.8)	48 (14.1)	83 (21.1)	<0.001
Inokin	189 (14.3)	61 (10.2)	58 (17.3)	70 (17.8)	<0.001
Hyde	313 (23.5)	158 (26.5)	65 (19.1)	90 (22.8)	0.118

Note. Percentages calculated out of all available responses. Missing data accounted for between 6% and 7% of each response and were omitted. Brand responses are not mutually exclusive. P-values (<0.05) indicate statistically significant trends in proportions across three age categories.

Table S4. Ever, past 30-, and past 7-day e-cigarette use by gender, age category, and device type, no. (%).

	13-20		21-24		25-40	
	Female	Male	Female	Male	Female	Male
**Ever Use**						
Any E-cigarette	**947 (48.6)**	553 (38.8)	452 (67.7)	210 (66.7)	463 (55.9)	**359 (61.7)**
Disposables	**737 (38.1)**	402 (28.4)	327 (49.4)	137 (43.9)	309 (37.6)	232 (40.2)
Pod/cartridge-based	**709 (36.7)**	414 (29.2)	337 (51.1)	162 (51.6)	335 (41.0)	**275 (47.8)**
Other E-cigarettes	**528 (27.3)**	328 (23.3)	283 (42.7)	129 (41.7)	291 (35.7)	226 (39.6)
**Past 30-Day Use**						
Any E-cigarette	**628 (32.2)**	388 (27.2)	358 (53.6)	161 (51.1)	330 (39.8)	**285 (49.0)**
Disposables	**456 (23.6)**	282 (19.9)	232 (35.0)	100 (32.1)	193 (23.5)	**174 (30.2)**
Pod/cartridge-based	**372 (19.3)**	228 (16.1)	225 (34.1)	116 (36.9)	215 (26.3)	**201 (35.0)**
Other e-cigarettes	274 (14.2)	172 (12.2)	181 (27.3)	94 (30.4)	188 (23.0)	**167 (29.2)**
**Past 7 Day Use**						
Any E-cigarette	**518 (26.6)**	326 (22.9)	229 (34.3)	**138 (43.8)**	273 (32.9)	**245 (42.1)**
Disposables	**358 (18.5)**	223 (15.7)	186 (28.1)	84 (26.9)	145 (17.7)	**142 (24.6)**
Pod/cartridge-based	283 (14.6)	178 (12.6)	168 (25.5)	93 (29.6)	169 (20.7)	**165 (28.7)**
Other e-cigarettes	192 (9.9)	140 (10.0)	141 (21.3)	72 (23.3)	139 (17.0)	**133 (23.3)**

Note. Bolded cells indicate statistically significant differences in proportions between males and females within each age category at $p < 0.05$.
